# Exploration of the Molecular Mechanisms Underlying the Anti-Photoaging Effect of *Limosilactobacillus fermentum* XJC60

**DOI:** 10.3389/fcimb.2022.838060

**Published:** 2022-04-29

**Authors:** Huizhen Chen, Ying Li, Xinqiang Xie, Moutong Chen, Liang Xue, Juan Wang, Qinghua Ye, Shi Wu, Runshi Yang, Hui Zhao, Jumei Zhang, Yu Ding, Qingping Wu

**Affiliations:** ^1^ School of Biology and Biological Engineering, South China University of Technology, Guangzhou, China; ^2^ Guangdong Provincial Key Laboratory of Microbial Safety and Health, State Key Laboratory of Applied Microbiology Southern China, Key Laboratory of Agricultural Microbiomics and Precision Application, Ministry of Agriculture and Rural Affairs, Institute of Microbiology, Guangdong Academy of Sciences, Guangzhou, China; ^3^ Department of Food Science and Technology, Institute of Food Safety and Nutrition, Jinan University, Guangzhou, China

**Keywords:** *Limosilactobacillus fermentum*, skin, anti-photoaging, nicotinamide, pan-genome analysis, UV

## Abstract

Although lactic acid bacteria (LAB) were shown to be effective for preventing photoaging, the underlying molecular mechanisms have not been fully elucidated. Accordingly, we examined the anti-photoaging potential of 206 LAB isolates and discovered 32 strains with protective activities against UV-induced injury. All of these 32 LABs exhibited high levels of 2,2-diphenyl-picrylhydrazyl, as well as hydroxyl free radical scavenging ability (46.89–85.13% and 44.29–95.97%, respectively). Genome mining and metabonomic verification of the most effective strain, *Limosilactobacillus fermentum* XJC60, revealed that the anti-photoaging metabolite of LAB was nicotinamide (NAM; 18.50 mg/L in the cell-free serum of XJC60). Further analysis revealed that LAB-derived NAM could reduce reactive oxygen species levels by 70%, stabilize the mitochondrial membrane potential, and increase the NAD^+^/NADH ratio in UV-injured skin cells. Furthermore, LAB-derived NAM downregulated the transcript levels of matrix metalloproteinase (*MMP*)-*1*, *MMP-3*, interleukin (*IL*)-*1β*, *IL-6*, and *IL-8* in skin cells. *In vivo*, XJC60 relieved imflammation and protected skin collagen fiber integrity in UV-injured Guinea pigs. Overall, our findings elucidate that LAB-derived NAM might protect skin from photoaging by stabilizing mitochondrial function, establishing a therotical foundation for the use of probiotics in the maintenance of skin health.

## 1 Introduction

Skin aging accompanies aging in humans, manifesting as roughness, sagging, fine lines, insufficient sweating, and increased sensitivity of the skin to changes in temperature (Giangreco et al., 2008). Both internal and external factors promote skin aging, and exposure to ultraviolet (UV) rays is one of the most common and preventable external factors affecting skin aging (Kammeyer and Luiten, 2015). Skin aging caused by UV exposure, also called photoaging, contributes to more than 80% of facial aging (Friedman, 2005; Han et al., 2014; Cavinato and Jansen-Dürr, 2017). Photoaging not only causes the edeterioration of appearance but also leads to skin barrier dysfunction and even cancer (Vicentini et al., 2011). Therefore, appropriate methods for preventing photoaging are urgently needed.

After exposure to UV radiation, the amorphous elastic fibers in the skin tissue exhibit excessive accumulation, whereas the collagen fibers appear to be abnormally broken and structurally disordered, gradually forming wrinkles in the skin (Varani et al., 2004; Watson et al., 2014). Moreover, when skin cells are exposed to excessive UV radiation, reactive oxygen species (ROS) can accumulate in the cells, leading to damage to skin cells and the extracellular matrix surrounding the cells (Morita, 2007; Giangreco et al., 2008). The pathophysiological mechanisms of photoaging are mainly related to ROS-induced damage, including activation of the mitogen-activated protein kinase signaling pathway and the phosphatidylinositol 3-kinase and nuclear factor-κB pathways and reductions in matrix metalloproteinase (MMP) synthesis and collagen production, ultimately promoting skin aging (Kammeyer and Luiten, 2015).

Skin health is not only related to skin cells but also profoundly influenced by the skin microbiome (Findley and Grice, 2014; Ladizinski et al., 2014), which is composed of millions of bacteria, fungi, and viruses. Similar to gut microbes, skin microbes play important roles in the defense against invasive pathogens, induction of immune responses, and decomposition of waste products (Scharschmidt and Fischbach, 2013; Grice, 2015; Ladizinski et al., 2014). Thus, microbiome treatment might be a novel solution for prevention of skin photoaging (Patra et al., 2020). Certain bacterial strains have major effects on improving skin conditions and preventing photoaging. For example, *Lactobacillus plantarum* HY7714 reduces skin collagen loss by affecting the activator protein-1 signaling pathway in skin cells (Kim et al., 2014; Lee et al., 2015). Further studies have suggested that the antiphotoaging mechanisms of *Lacticaseibacillus rhamnosus* GG (ATCC 53103, LGG) and *Lacticaseibacillus casei* strain Shirota might be related to their antioxidant properties (Yau et al., 2020; Mai et al., 2021). Taken together, these studies have demonstrated the great potential of microbiome treatment for the alleviation of photoaging in the skin. However, the exact metabolites of probiotic strains and the molecular mechanisms through which skin microbes affect photoaging have not been fully elucidated, and the applications of microbiome treatments remain limited. Herein, we explored the molecular mechanisms through which lactic acid bacteria (LAB) exert antiphotoaging effects to provide a theoretical basis for the application of probiotics in skin health care.

## 2 Materials and Methods

### 2.1 Screening of LABs With Anti-Photoaging Activity Against UVB

#### 2.1.1 Bacterial Isolation and Phylogenetic Analysis

We isolated 186 LAB strains from fermented foods and animal feces collected in the Xinjiang Uygur Autonomous Region and 16 LAB strains from the feces of healthy centenarians in Guangdong, China using the methods described by Zeng (Zeng et al., 2020). In addition, four standard strains (ATCC53103, ATCC7469, ATCC393, ATCC14917) were investigated as controls. Studies have demonstrated the antioxidant properties of these four strains, and they were applied to improve various diseases (Lin et al., 2009; Zahran et al., 2017; Mantzourani et al., 2018; Xu et al., 2018; Liu et al., 2021).

Genomic DNA from all LAB was extracted using a genomic DNA extraction kit (Magen Biotech, Guangzhou, Guangdong, China). LAB species were identified by an evaluation of 16S rRNA gene sequences. The 16S rRNA gene was amplified using universal primers (27F, 5′-AGAGTTTGATCCTGGCTCAG-3′; 1492R, 5′-ACGGCTACCTTGTTACGACTT-3′), as described by Garrity (Garrity, 2016). The polymerase chain reaction products were sent to GENEWIZ (Suzhou, China) for Sanger sequencing, and the results were compared with reference sequences from GenBank (http://www.ncbi.nlm.nih.gov/BLAST) to confirm the LAB taxonomy.

#### 2.1.2 Cell Culture

HaCaT primary human keratinocytes were obtained from Dr. Chen (First Affiliated Hospital of Sun Yat-sen University) and maintained at 37°C in a 5% CO_2_ incubator (ThermoFisher Scientific, Waltham, MA, USA) in complete medium containing Dulbecco’s modified Eagle’s medium (ThermoFisher Scientific) with 10% (v/v) fetal bovine serum (Gibco, NY, USA) and antibiotics-antimycotic (100 U penicillin, 100 μg/mL streptomycin, and 0.25 μg/mL amphotericin B; HyClone, UT, USA).

#### 2.1.3 Minimum Nontoxic Dilution (MNTD) Preparation

All LAB were grown in de Man Rogosa Sharpe (MRS) broth (HuanKai Microbial, Guangzhou, Guangdong, China) at 37°C for 48 h in an anaerobic workstation (Don Whitley Scientific, W Yorkshire, UK). The cells were pelleted by centrifugation at 10,000 × *g* at 4°C for 10 min, and supernatants were collected using 0.22 μm microfilters (HuanKai Microbial). Cell-free supernatants (CFSs) and uninoculated MRS broth controls were adjusted to pH 7.35–7.45 using sodium hydroxide (Sigma-Aldrich, St. Louis, MO, USA) and stored at −80°C until use. The cytotoxicity of the pH-adjusted CFS samples was assessed using Cell Counting Kit-8 (CCK-8) assays (GLPBIO, Montclair, CA, USA) according to the manufacturer’s instructions. Five-fold dilutions of CFSs were generated, and the cytotoxicity of each CFS was re-evaluated if HaCaT cell viability failed to remain at 100% after 24 h of incubation with 10% CFSs in complete cell medium. The CFSs with the lowest toxicity were defined as the MNTDs.

#### 2.1.4 UVB Exposure in HaCaT Cells

HaCaT cells were seeded in 96-well plates at a density of 1 × 10^5^ cells/well and grown for 24 h to reach 80% confluency. After replacing cell supernatants with 100 μL phosphate-buffered saline, HaCaT cells were exposed to UVB at 18 mJ/cm^2^ for 5 min using a UVB-313EL light tube (ANTOINE, Guangzhou, Guangdong, China). The supernatants were changed to 100 μL complete cell medium after UV radiation for subsequent studies.

#### 2.1.5 High-Throughput Assay to Screen for the Anti-UVB Potential of MNTDs

The viability of HaCaT cells treated with MNTDs of LAB at 24 h after UVB exposure was examined to screen for the antiphotoaging potential of LAB. Briefly, the viability of UVB-exposed cells was tested after incubation for 24 h with 10% MNTDs in complete cell medium using a CCK-8 kit (GLPBIO). pH-adjusted MRS-treated cell suspensions were used as a positive control, and complete cell medium was used as a negative control. Absorbance at 450 nm was then quantified with a microplate reader (Biotek, Winooski, VT, USA).

### 2.2 Evaluation of Antioxidant Effects of LABs

#### 2.2.1 Free Radical Scavenging Ability

Because antioxidant ability can be the main reason for the antiphotoaging effects of LAB (Han et al., 2014), we evaluated the 2,2-diphenyl-picrylhydrazyl (DPPH) and hydroxyl radical-scavenging activities of the MNTDs for 206 LAB strains. The method of Shimada was used to assess the DPPH free radical-scavenging ability of the MNTDs of different LAB. Briefly, 100 μL MNTDs was added to 100 μL of 0.2 mM methanolic solution of DPPH (Yuanye Biology, Shanghai, China) and incubated in the dark at room temperature for 30 min. The absorbance of the resulting solution was measured at 517 nm with a microplate reader (Biotek). Distilled water was used as a control in the test. The DPPH free radical-scavenging activity was calculated using the following formula.


$$DPPH\;radical\;scavenging\;capability(\%)=\frac{A_{C}-A_{S}}{A_{C}}\times 100\;(1)$$

In this formula, A_s_ is the absorbance value of MNTDs, and A_c_ is the absorbance value of the control.

The scavenging ability of hydroxyl radicals was determined using the method described by Liu (Liu et al., 2009). A mixture of 30 μL of 0.75 mM 1,10-phenanthroline (MACKLIN, Shanghai, China), 30 μL of 0.75 mM FeSO_4_ (Chemical Reagent, Guangzhou, Guangdong, China), 30 μL of 0.01% (v/v) H_2_O_2_, 60 μL of 0.2 M (pH 7.4) sodium phosphate buffer, and 30 μL MNTDs was incubated at 37°C for 60 min, and the absorbance of the mixture was measured at 536 nm with a microplate reader (Biotek).


$$Hydroxyl\;radical\;scavenging\;activity\;(\%)=\frac{A_{S}-A_{C}}{A_{0}-A_{C}}\times 100\;(2)$$

In this formula, A_0_ is the absorbance of deionized water instead of H_2_O_2_, A_S_ is the absorbance value of CFS, and A_C_ is the absorbance without CFS.

#### 2.2.2 ROS Generation Assay

Intracellular ROS levels were measured using a Reactive Oxygen Species Assay Kit (Beyotime Biotechnology, Shanghai, China). Briefly, the cells were seeded in 6-well plates and exposed to UV radiation as described. The cells were then treated with 10% MNTDs from different LAB. Following treatment, the cells were incubated with DCFH-DA for 20 min at 37°C in the dark, and fluorescence was measured using a BD FACSCanto II Flow Cytometer (BD Biosciences, San Jose, CA, USA). pH-adjusted MRS-treated cell suspensions were chosen as the negative control. Fluorescence images were acquired using a CYTATION 5 Imager Reader (Biotek).

### 2.3 Genomic Mining of the Anti-Photoaging Metabolites of LABs

#### 2.3.1 Genomic DNA Library Preparation

Genomic DNA libraries were constructed using AMT Rapid DNA-Seq Kits for Illumina (CISTRO, Guangzhou, Guangdong, China), with fragmentation, end-repair, adaptor ligation with Illumina adapters, size selection with beads, and library DNA amplification; all methods were performed according to the manufacturer’s instructions. The libraries were assessed using an Agilent Bioanalyzer 2100 (Agilent Technologies, Santa Clara, CA, USA) and a Qubit 3.0 fluorometer (Invitrogen, Carlsbad, CA, USA). DNA sequencing was performed on an Illumina Nextseq 550 platform (Illumina, San Diego, CA, USA) with a High Output v2.5 kit (Illumina). Long reads of microbial genomic DNA libraries were prepared using a Rapid Barcoding Sequencing Kit (Nanopore, Oxford, UK) and sequenced on a Nanopore MinION platform with R9.4.1 flow cells (Nanopore).

Low-quality reads from Illumina sequencing were filtered out using Trimmomatic software (v0.39) (Bolger et al., 2014). Low-quality and short reads from Nanopore sequencing were filtered using Filtlong software (v0.2.0; https://github.com/rrwick/Filtlong). The filtered Illumina and Nanopore reads were aligned into *de novo* assembled contigs using Unicycler software (v0.4.8) (Nurk et al., 2013).

#### 2.3.2 Genome Annotations and Comparative Genomics Analysis

Pan-genome analysis was performed on the Prokka output using Roary (v3.11.2) with a BLASTP identity cut-off of 95% (Page et al., 2015). The LAB strain core-genome was produced using Harvest software (v1.1.2), with ATCC 14931 as the reference genome (Treangen et al., 2014). Following core-genome alignment, Gubbins was used for recombination analysis and the removal of putative recombined regions (Croucher et al., 2015).

#### 2.3.3 Nicotinamide Quantitation

A Shimadzu high-performance liquid chromatography (HPLC) system (Shimadzu, Tokyo, Japan), equipped with an LC-20A UV detector (Shimadzu), was used with a COCOSMOSIL 5C18-PAQ column (5 μm, 4.6 mm × 250 mm) for the separation of compounds. The temperature of the column compartment was maintained at 30°C throughout the analysis. The wavelength of detection was 254 nm, and the injection volume was 20 μL. All chromatographic assays were performed with a flow rate of 1 mL/min, and 25 mM potassium dihydrogen phosphate buffer was used as the mobile phase. The method was validated by checking the accuracy, precision, linearity, limit of detection, limit of quantitation, and specificity prior to sample analysis.

### 2.4 Study of the Anti-Photoaging Mechanism of *L. fermentum* XJC60 and Nicotinamide

#### 2.4.1 Measurement of Mitochondrial Membrane Potential

Intracellular mitochondria membrane potential levels were measured using a Mitochondria Membrane Potential Kit (Sigma-Aldrich). Briefly, the cells were collected from the plates and incubated with 500 µL JC-10 Dye Loading Solution for 15 min at 37°C in the dark. Fluorescence was measured using a BD FACSCanto II Flow Cytometer (BD Biosciences). pH-adjusted MRS-treated cell suspensions were used as the negative control.

#### 2.4.2 Analysis of NAD^+^/NADH Levels

After treatment of HaCaT cells (1 × 10^6^ cells/sample), cells were collected, and intracellular NAD^+^ levels were determined using an NAD^+^/NADH assay kit with WST-8 (Beyotime Biotechnology). Briefly, cells were lysed with 200 μL cold lysis buffer. To measure total NAD^+^ and NADH concentrations, 20 μL of cell lysate was added to each well of a 96-well plate. To measure NADH levels, lysed cell suspensions were incubated at 60°C for 30 min, and 20 μL was then added to each well of a 96-well plate. Subsequently, 90 μL alcohol dehydrogenase was added to each well, and the plates were incubated at 37°C for 10 min. Finally, 10 μL chromogenic solution was added, and the mixture was incubated at 37°C for 30 min. A standard curve was generated and measured at the same time as the samples. The absorbance values were measured at 450 nm and analyzed on a microplate reader (Biotek). The amount of NAD^+^ was derived by subtracting NADH from total NAD^+^/NADH. pH-adjusted MRS-treated cell suspensions were used as the negative control.

### 2.5 Evaluation of the Anti-Photoaging Effect of *L. fermentum* XJC60 and Nicotinamide *In Vitro*

#### 2.5.1 Enzyme-Linked Immunosorbent Assay (ELISA)

After treating HaCaT cells (1 × 10^6^ cells/sample), cells were collected, and MMP-1 concentrations were determined using an MMP-1 ELISA kit (Bosterbio, Pleasanton, CA, USA) according to the manufacturer’s protocol. pH-adjusted MRS-treated cell suspensions were used as the negative control.

#### 2.5.2 Reverse Transcription Polymerase Chain Reaction (RT-PCR)

After treating HaCaT cells (1 × 10^6^ cells/sample), cells were collected, and RNA was extracted using a HiPure Total RNA Mini Kit (Magen). cDNA was synthesized from 1 µg of total RNA using Evo M-MLV reverse transcriptase (Accurate Biology AG, Changsha, Hunan, China) according to the manufacturer’s instructions. Primers for *MMP-1*, *MMP-3*, interleukin (IL)-1β, *IL-6*, *IL-8*, and glyceraldehyde 3-phosphate dehydrogenase (*GAPDH*), which were used for semiquantitative RT-PCR (**Supplementary Table S1**), were synthesized by GENEWIZ. The qPCR assay was performed using a LightCycler96 (Roche Diagnostics Corporation, Indianapolis, IN, USA) using the following conditions: 95°C for 30 s; 40 cycles of 95°C for 5 s, 60°C for 30 s, 95°C for 5 s, 60°C for 60 s, and 95°C for 1 s. Data were analyzed using LightCycler96 SW software (Roche Diagnostics Corporation).

### 2.6 Evaluation of the Anti-Photoaging Effect of *L. fermentum* XJC60 *In Vivo*

#### 2.6.1 Experimental Animals and Topical Administration

Six female Dunkin Hartley Guinea pigs (weight: 250–350 g) were purchased from the Laboratory Animal Center of Southern Medical University (Guangzhou, Guangdong, China) and were allowed to acclimate for 1 week before the experiment. The animals were housed in a climate-controlled facility with a temperature of 24°C, humidity of 50%, dark:light cycle of 12:12 h, and free access to food and water. All experimental protocols were approved by the Institutional Animal Care and Use Ethics Committee of Institute of Microbiology, Guangdong Academy of Science (approval no. GT-IACUC202010225).

The methods used for the animal experiment were obtained from the Cosmetic Safety Technical Specification. Briefly, 18–24 h prior to the UVB damage test, the back skin of each animal was depilated. The skin at the test site was intact and free of damage. The depilated skin was divided into four zones, each measuring approximately 2 cm × 2 cm. The four zones were treated as follows: zone 1 was the test group (0.2 mL MNTDs from *Limosilactobacillus fermentum* XJC60 [n = 6] was applied externally after UVB irradiation [75 mJ/cm^2^]); zone 2 was the control group (0.2 mL pH-adjusted MRS [n = 6] was applied externally after UVB irradiation [75 mJ/cm^2^]); and zones 3 and 4 were the blank control group without UVB radiation but treated with 0.2 mL *L. fermentum* XJC60 or pH-adjusted MRS externally.

#### 2.6.2 Histological Analyses

Histological analyses were performed on hematoxylin/eosin (H&E)-stained skin samples fixed in 4% paraformaldehyde, embedded in paraffin, and stained using an H&E Stain Kit (Solarbio Life Science, Beijing, China). Images were captured using a flat-panel microscope for analysis (Kangtao Technology, Wuhan, Hubei, CHINA).

### 2.7 Statistical Analysis

Data analysis was performed using t-tests or by one-way analysis of variance with GraphPad Prism Software (GraphPad, La Jolla, CA, USA). Results with *P* values of less than 0.05 were considered statistically significant. Data are presented as means ± standard deviations (SDs) of at least three independent experimental replicates.

## 3 Results

### 3.1 High-Throughput Screening of the Antiphotoaging Potential of LAB Strains

**Figure 1:**
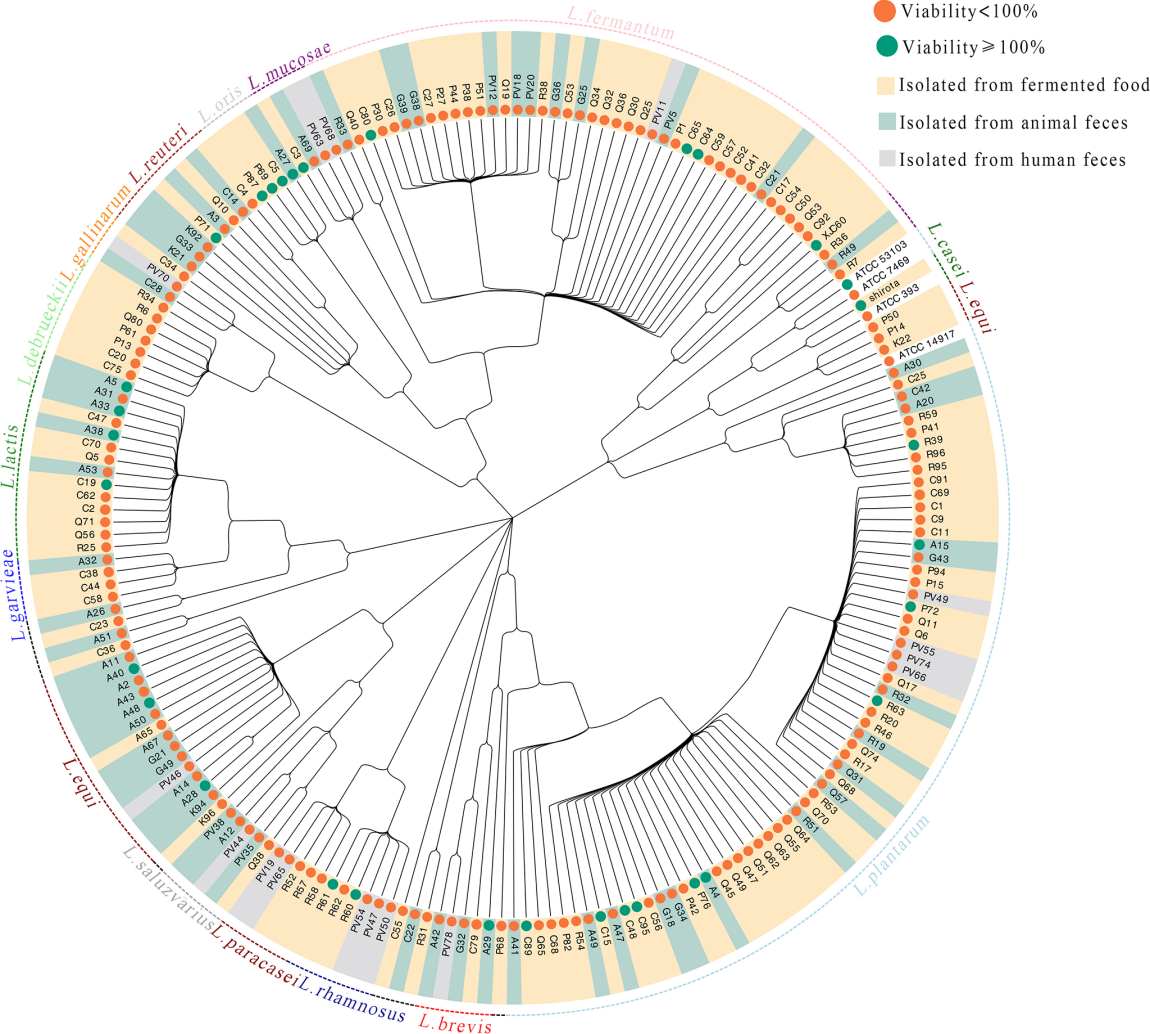
Evolutionary tree of the lactic acid bacteria (LAB) isolates and their abilities to repair UVB damage The evolutionary tree was built according to the 16S rRNA gene sequences of 206 strains of LAB. The taxonomy of each LAB strain is shown in the outer circle of the tree, and isolation information for each LAB strain is indicated by the bar color. The antiphotoaging potential (cell viability protection) for each strain is indicated using a green or orange node.

The 206 strains included in this study comprised 16 different LAB species (**Figure 1**). The viabilities of UVB-injured cells are depicted using different node colors. Notably, the MNTDs of 32 LAB strains showed strong recovery activities for UVB-injured HaCaT cells, and cell viability increased from 50% to 100% after UVB irradiation (*P* < 0.05 compared with MRS-treated cells; **Supplementary Table S2**).

### 3.2* In Vitro* Assessment of the Antioxidant Effects of LAB Strains

**Figure 2:**
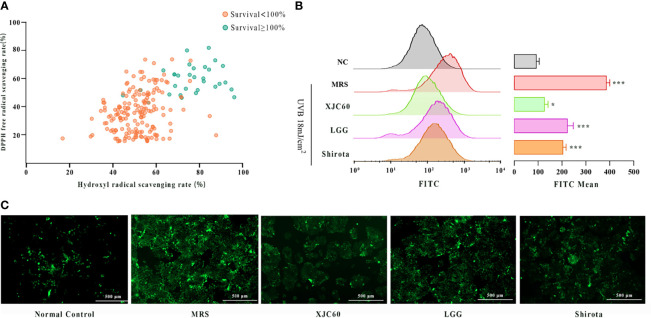
*In vitro* antioxidant effects of lactic acid bacteria (LAB) strains. **(A)** DPPH and hydroxyl radical-scavenging activities of minimum nontoxic dilutions (MNTDs) for the 206 LAB strains. **(B)** Reactive oxygen species (ROS) levels of different MNTDs in UVB-injured HaCaT cells, as examined by flow cytometry. **(C)** Fluroescence images of ROS staining for MNTD-treated UVB-injured HaCaT cells. All data are presented as means ± SDs (n = 3). *P < 0.05, ***P < 0.001 compared with the normal control (NC) group.

To investigate whether the MNTDs of LAB strains could repair skin cells by reducing UVB-induced oxidative stress in HaCaT cells, we assessed the DPPH and hydroxyl radical-scavenging activities of the 206 LAB strains *in vitro*. The results revealed that 32 strains providing strong protection against UVB injury had stronger antioxidant activities than those without significant protection for both DPPH and hydroxyl radicals (**Figure 2A**). Among these strains, we found that *L. fermentum* XJC60 showed the best antioxidative effects, with a DPPH free radical-scavenging rate of 85.13% and hydroxyl free radical-scavenging rate of 84.14%. We further explored the antioxidative activity of this strain using UVB-irradiated HaCaT cells. Flow cytometric analysis revealed that ROS generation by HaCaT cells was increased by UVB radiation, and the FITC channel number increased from 99.23 ± 11.65 to 404.70 ± 14.01 (*P* < 0.001). Additionally, treatment at the MNTDs of *L. fermentum* XJC60 significantly decreased ROS levels to a mean FITC channel number value of 134.70 ± 14.50 (*P* = 0.030; **Figures 2B, C**). Further analysis showed that the antioxidative activity of the MNTD of *L. fermentum* XJC60 was stronger than that of *L. rhamnosus* GG (ATCC 53103, LGG) and *L. casei* strain Shirota, two universally recognized strains with strong antioxidant activities (Lin et al., 2009; Finamore et al., 2018; Liu et al., 2021), indicating that *L. fermentum* XJC60 was a unique isolated strain with great potential in antiphotoaging effects.

### 3.3 Genomic Mining of Antiphotoaging Metabolites of LAB

**Figure 3:**
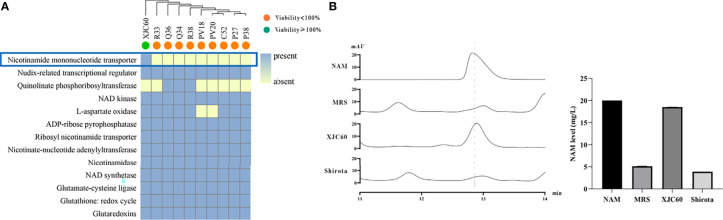
Genomic mining and validation of the antiphotoaging metabolites of lactic acid bacteria (LAB). **(A)** Genes involved in the NAD, NADP, and oxidative stress pathways in different LAB strains. The antiphotoaging potential is indicated as a node, with green representing high potential and orange representing low potential. The existence of a gene is shown in the squares, with blue representing the presence of the gene and yellow representing the absence of the gene. **(B)** HPLC quantification of nicotinamide (NAM) levels in the minimum nontoxic dilutions (MNTDs) of different LAB strains.

To identify antiphotoaging metabolites in LAB, we applied pan-genomic analysis to identify the unique genes in LAB strains with high antioxidant activities. Pan-genome analysis revealed that *L. fermentum* XJC60 shared 1436 core genes (> 99% presence) with 10 *L. fermentum* isolates in our study. Among them, we identified nicotinamide mononucleotide transporter, which is associated with antiphotoaging functions, as a strain-specific gene in *L. fermentum* XJC60. This gene has been reported to contribute to the generation of nicotinamide (NAM) synthesis in microorganisms (Poddar et al., 2019), and its existence in the genome suggested the presence of high levels of NAM in the MNTDs of *L. fermentum* XJC60 (**Figure 3A**).

To validate our hypothesis, we further examined the NAM levels in the MNTDs of LAB. Our results showed that the NAM level in the MNTD of *L. fermentum* XJC60 was 18.50 ± 0.01 mg/L, which was much higher than that (5.14 ± 0.01 mg/L) in pH-adjusted MRS broth (*P* < 0.001). Further analyses revealed that the NAM level of *L. fermentum* XJC60 was also higher than that (3.91 ± 0.01 mg/L) of *L. casei* strain Shirota (*P* < 0.001; **Figure 3B**).

### 3.4 Evaluation of the Mitochondrial Protective Effects of *L. fermentum*-Derived NAM

**Figure 4:**
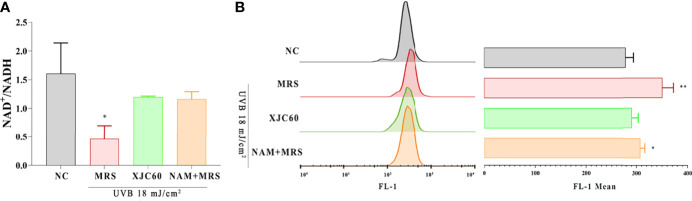
Evaluation of mitochondrial protection by *Limosilactobacillus fermentum*-derived nicotinamide (NAM). **(A)** Effects of minimum nontoxic dilutions (MNTDs) on the NAD^+^/NADH ratio in *L. fermentum* XJC60 and equal amounts of NAM on UVB-injured HaCaT cells. **(B)** Flow cytometric analysis of JC-1 levels with different MNTDs in LAB. Data are presented as means ± SDs (n = 3). *P < 0.05, **P < 0.01 compared with the NC group.

ROS are mainly produced in mitochondria, and changes in the mitochondrial membrane potential and NAD^+^/NADH levels are closely related to the state of mitochondria (Birch-Machin, 2000; Naidoo et al., 2018). The detection of JC-1-loaded cells in the FL-1 channel is commonly used to detect mitochondrial membrane potential (Elefantova et al., 2018). To validate the antioxidant functions of L. fermentum-derived NAM, we examined the JC-1 level the and NAD^+^/NADH ratio in UVB-injured HaCaT cells. Our results revealed that treatment with the MNTD of L. fermentum XJC60 increased the NAD^+^/NADH ratio by 2-fold (P = 0.027; **Figure 4A**) and downregulated the JC-1 level from a mean FL-1 channel value of 348.00 ± 21.52 to 288.70 ± 12.50 (P = 0.014; **Figure 4B**). Our results also revealed that pH-adjusted MRS containing a similar concentration (18.50 mg/L) of NAM had the same protective effects on mitochondria. There was no significant difference between NAM-containing MRS and the MNTD of L. fermentum XJC60 both in the NAD^+^/NADH ratio and JC-1 level (1.16 ± 0.13 versus 1.20 ± 0.02 for the NAD^+^/NADH ratio, P = 0.661; mean FITC channel value of 305.30 ± 8.74 versus 288.70 ± 12.50 for the JC-1 level, P = 0.131; **Figure 4**). Taken together, these results indicated that LAB-derived NAM is an essential metabolite mediating antiphotoaging activity in UVB-injured HaCaT cells.

### 3.5 *In Vitro* Assessment of the Antiphotoaging Potential of LAB-Derived NAM

**Figure 5:**
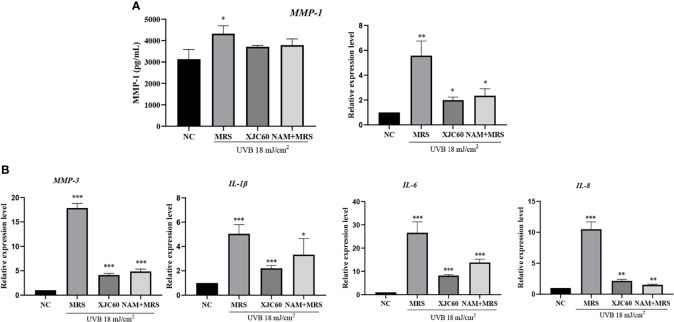
Effects of *Limosilactobacillus fermentum* XJC60 and nicotinamide (NAM) on ECM and inflammation in UVB-injured HaCaT cells. **(A)** Evaluation of MMP-1 protein and mRNA levels. **(B)** mRNA levels of other MMPs and inflammation-related factors. Relative mRNA levels of *MMP-1*, *MMP-3*, *IL-1β*, *IL-6*, and *IL-8* in UVB-injured HaCaT cells were calculated using qPCR, with normalization to *GAPDH* expression. Data are presented as means ± SDs (n = 3). *P < 0.05, **P < 0.01, ***P < 0.001 compared with the normal control (NC) group.

Wrinkles and roughness of photoaged skin are mainly related to degradation and inflammation of skin cells (Makrantonaki et al., 2013). Therefore, we further analyzed the antiphotoaging effects of LAB-derived NAM. ELISA showed that LAB-derived NAM effectively reduced the amount of MMP-1 from 4323.00 ± 366.90 pg/mL to 3712.00 ± 63.06 pg/mL (P = 0.020), and similar changes were observed for NAM-containing MRS (3786.00 ± 290.30 pg/mL, P = 0.010; **Figure 5A**).

We also evaluated the antiphotoaging potential of LAB-derived NAM at the transcript level in HaCaT cells. Our results showed MMP-1 mRNA levels were significantly downregulated by 14.02-fold by the MNTD of L. fermentum XJC60 (P = 0.002). Additionally, MMP-3, IL-1β, IL-6, and IL-8 were downregulated by 4.32-fold (P = 0.001), 2.29-fold (P < 0.001), 3.24-fold (P < 0.001), and 4.89-fold (P = 0.001), respectively. We also examined the antiphotoaging effects of NAM-containing MRS and found similar changes in the mRNA levels of MMPs and inflammation-related factors (P > 0.05 compared to those with the MNTD of L. fermentum XJC60). Together, these results indicated that the NAM produced by L. fermentum exerted strong antiphotoaging effects through the inhibition of MMPs and inflammation in UVB-injured keratinocytes (**Figure 5B**).

### 3.6 *In Vivo* Antiphotoaging Effects of *L. fermentum* XJC60

**Figure 6:**
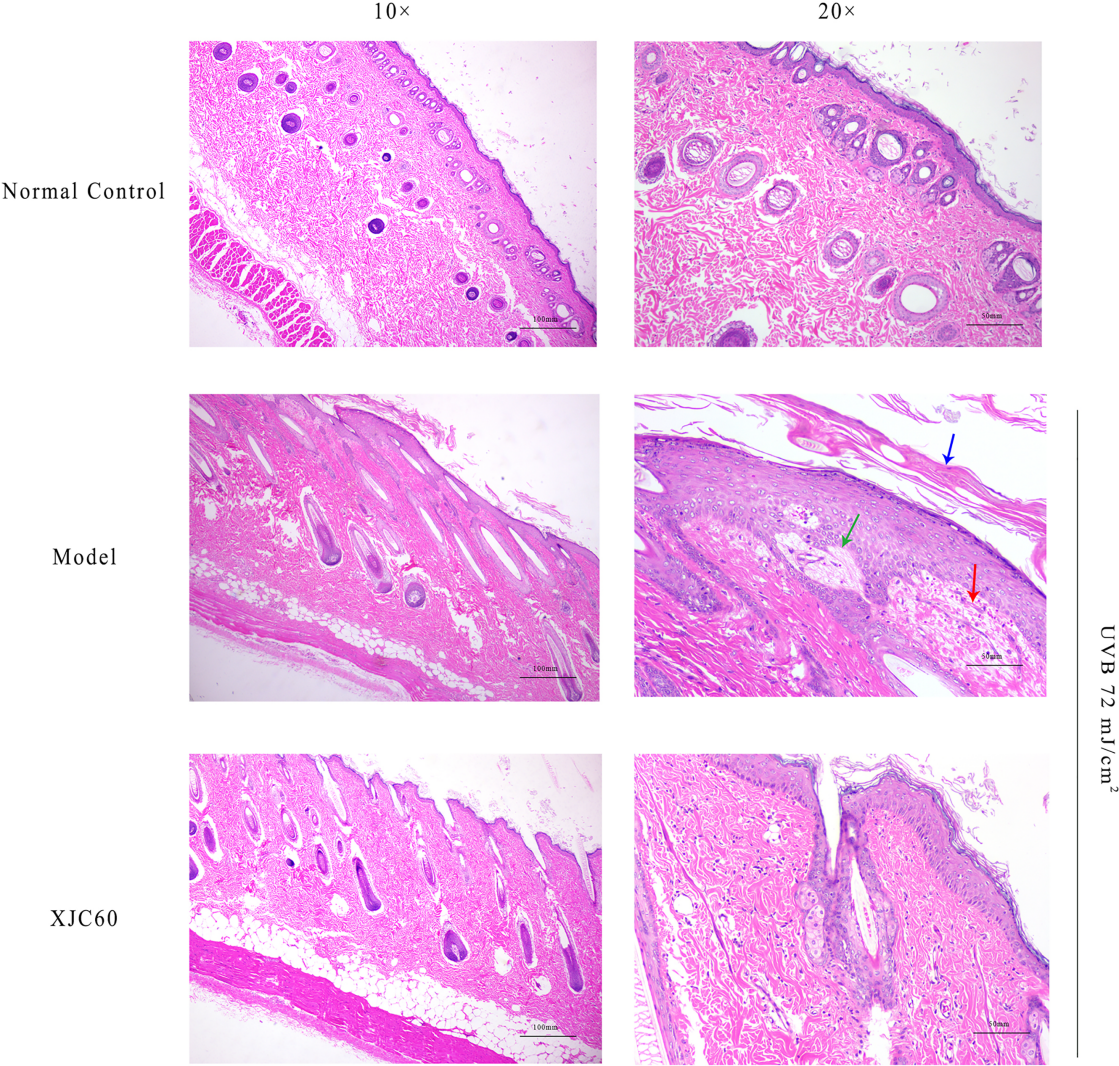
*In vivo* antiphotoaging effects of *Limosilactobacillus fermentum* XJC60. (Normal control) Left: smooth stratum corneum and obvious keratinized beads (10×); right: no inflammatory cell infiltration or cell necrosis was detected (20×). (Model) Left: abnormal keratinization in the skin of Guinea pigs, with obvious proliferation (10×); right: visible epithelial edema and mild cell necrosis (20×). Blue arrow: keratinization; green arrow: epithelial edema; red arrow: cell necrosis. (*L. fermentum* XJC60) Left: the skin tissue structure was relatively complete, keratinization of the skin was reduced, and keratinization beads were being repaired (10×); right: no inflammatory cell infiltration or cell necrosis were observed (20×).

To evaluate the antiphotoaging effects of the MNTD of L. fermentum XJC60, pathological changes were investigated in H&E-stained skin sections. Compared with that in non-irradiated tissues, UVB radiation induced abnormal keratinization in the skin of Guinea pigs, with obvious proliferation, visible epithelial edema, and mild cell necrosis (**Figure 6**). Furthermore, the topical application of the MNTD of L. fermentum XJC60 significantly alleviated UVB damage, which was characterized by a complete skin tissue structure, orderly arrangement of the dermis and epidermis, and abundant and complete subcutaneous hair follicles and sebaceous glands (**Figure 6**). This also confirmed the previous results in section 3.5; specifically, the external application of the MNTD of L. fermentum XJC60 reduced the high MMP-1 levels in epidermal cells caused by UVB irradiation, and thus, the abnormal keratinization and hyperplasia of the Guinea pig epidermis was improved. In addition, there was no edema and inflammatory cell infiltration in the XJC60 group, which was attributed to the decreased expression of inflammatory factors in the skin of Guinea pigs induced by the MNTD of L. fermentum XJC60.

## 4 Discussion

With the development of next-generation sequencing, we found that skin microbes play vital roles in skin function, thereby affecting the health of the skin and even the whole body (Scharschmidt and Fischbach, 2013). In fact, research showed the species richness of the skin microecology gradually increases with age, hinting at a correlation between the skin microbiota and aging (Ying et al., 2015; Li et al., 2020; Luna, 2020). However, the specific mechanisms through which the skin microbiome mediates skin aging remain unclear, and further studies at the cellular level are needed.

Photoaging is caused by oxidative damage and decreased collagen synthesis in skin cells after exposure to UV radiation (Kammeyer and Luiten, 2015). Previous studies have demonstrated that photoaging is related to certain microorganisms, and probiotics can have antiphotoaging effects (Li et al., 2020). For example, *Lactobacillus acidophilus* KCCM12625 can significantly suppress ROS generation in UVB-injured skin cells, thereby reducing the photoaging phenomenon caused by oxidative damage (Lim et al., 2020). However, the molecular mechanisms mediating the antiphotoaging effects of probiotics have not yet been thoroughly analyzed.

Multiomics-based microbial function mining provides a new approach for the exploration of molecular mechanisms. For example, Lloyd-Price used a multiomics database to explore the relationships between intestinal microbial activities and inflammatory bowel disease and explained the key functions of acylcarnitines from *Clostridia* in disease progression (Lloyd-Price et al., 2019). Such studies have illustrated that the key factors through which the microbiome affects human health are active microbiologic metabolites (Koh et al., 2016). Therefore, this strategy might be a practical approach for an analysis of the functions of key microbial metabolites in human health, and we expect that there could be a similar mechanism between skin microecology and photoaging.

In this study, we combined pan-genomic and metabonomic methods to explore the key metabolites of probiotics that protect against photoaging and their mechanisms of action. We tested 206 LAB isolates from different sources and found that 32 isolates had protective effects on UVB-damaged skin cells, suggesting potential applications in the prevention of photoaging (**Figure 1**). Furthermore, the results revealed that 32 strains providing protection against UVB injury had stronger antioxidant activities than those without significant protective effects from radicals (**Figure 2A**). The strong anti-photoaging potential of *L. fermentum* XJC60 significantly reduced the high ROS level in UVB-damaged HaCaT cells (**Figures 2B, C**).Therefore, we found that a reduction in intracellular oxidative stress was the main antiphotoaging mechanism of LAB. Indeed, LAB fermentation broth contained a variety of antioxidant factors, which could quickly degrade free radicals and hydroxyl groups, reduce intracellular ROS production, and repair photoaging cells.

Furthermore, we used pan-genomics combined with metabolomics to identify key metabolites mediating the antiphotoaging effects of LAB. Data mining revealed a strain-specific protein, nicotinamide mononucleotide transporter, which promoted antioxidant activity in *L. fermentum* XJC60 (**Figure 3A**). Nicotinamide mononucleotide transporter can directly transport extracellular NMN into the cell and increase the level of NAM, thereby regulating NAD^+^ metabolism (Wu and Sinclair, 2019; Lloyd-Price et al., 2019; Yachida et al., 2019). We further quantified the LAB metabolites by HPLC and found high levels of NAM (18.50 mg/L) in culture broth from the MNTD of *L. fermentum* XJC60 (**Figure 3B**), validating the pan-genomics results. Previous studies have demonstrated the protective effects of NAM on photoaging, and clinical trials have demonstrated that oral nicotinamide can prevent UV-induced immunosuppression and photocarcinoma (Yiasemides et al., 2009; Park et al., 2010; Thompson et al., 2015). In particular, this is the first study demonstrating that NAM produced by LAB also exhibit strong antiphotoaging effects.These results suggest that XJC60 might repair the oxidative stress state through the NAD^+^ pathway, thereby maintaining the normal homeostasis of UV-damaged skin cells and ultimately delaying skin photoaging.

As part of glycolysis and the tricarboxylic acid cycle, NAD^+^ is an important cofactor for cell respiration metabolism, and the NAD^+^/NADH level affects the normal progression of complex I in the mitochondrial respiratory chain (Fania et al., 2019). If the level of NAD^+^/NADH is too low, the mitochondrial respiratory chain will be disrupted, and the production of ATP will halt owing to impairments in calcium flow in the cell; this then reduces mitochondrial membrane potential and blocks mitochondrial function (Brand et al., 2018; Owens et al., 2013). Our findings also showed that LAB-derived NAM could significantly increase the levels of NAD^+^/NADH and recover mitochondrial membrane potential in UVB-injured HaCaT cells (**Figure 4**). Because the mitochondrial respiration program damaged by UVB was repaired, the energy-dependent DNA repair mechanism could be restored to normal (Holmström and Finkel, 2014). Therefore, produced as a by-product of the respiratory chain, the ROS level was also reduced, the oxidative stress in skin cells was alleviated, and cell viability returned to normal (**Figures 1**, **2**). In addition, UV-induced ROS stimulates the synthesis of MMPs and pro-inflammatory factors, leading to the degradation of collagen and typical symptoms of inflammation and dry, peeling skin (Scharffetter-Kochanek et al., 2000). In our study, LAB-derived NAM reduced the expression levels of MMPs and ILs in UVB-injured HaCaT cells and prevented abnormal keratinization, epithelial edema, and inflammatory cell infiltration *in vivo* (**Figures 5**, **6**).

However, there were still some limitations to our study. Although our results indicated that *Lactobacillus*-derived NAM is an important metabolite protecting against photoaging in *L. fermentum* XJC60, we also found that its MNTDs resulted in stronger protection for skin cells than a similar dose of NAM. This result suggested that there might be some other active substances in *L. fermentum* XJC60 that should be explored. In addition, we only evaluated the functions of LAB-derived metabolites; the effects of live bacteria on UV-damaged skin have not yet been elucidated. Therefore, further studies are needed to assess the interactions between LAB and UV-damaged skin.

## 5 Conclusions

In this study, we combined pan-genomics and metabolomics to identify new antiphotoaging factors from metabolites of LAB strains with antiphotoaging potential. We discovered that the NAM mononucleotide transporter produced by LAB was beneficial for the synthesis of NAM, which might be a key metabolite for skin protection. Furthermore, we demonstrated that LAB-derived NAM could stabilize mitochondrial function and reduce ROS generation in UVB-injured skin cells, thereby suppressing collagen degradation and inflammation *in vitro* and *in vivo*. In addition, our results suggested that there might be some other active substances in LAB, which should be explored further. Overall, our findings provided insights into the antiphotoaging mechanisms of probiotics and established novel approaches for the maintenance of skin health using LAB-derived NAM as an antiphotoaging treatment.

## Data Availability Statement

The datasets presented in this study can be found in online repositories. The name of the repository and accession numbers can be found below: NCBI; PRJNA703332, PRJNA703368, PRJNA703369, PRJNA703370, PRJNA703371, PRJNA703372, PRJNA703373, PRJNA703374, PRJNA703375, PRJNA703376.

## Ethics Statement

The animal study was reviewed and approved by the Institutional Animal Care and Ethics Committee of Institute of Microbiology, Guangdong Academy of sciences.

## Author Contributions

Conceptualization, HC, YL, XX, and QW. Methodology, HC, YL, LX, QY, and RY. Software, HC, YL, XX, LX, and SW. Validation, HC, YL, MC, LX, HZ, and MC. Formal analysis, HC, YL, MC, and YD. Investigation, HC, YL, LX, JW, and XX. Resources, XX, JW, JZ, and YD. Data curation, YL and XX. Writing—original draft preparation, HC and YL. Writing—review and editing, XX, QY, HZ, RY, YD, and QW. Visualization, HC, SW, and JW. Supervision, XX, YD, and QW. Project administration, JZ, YD, and QW. Funding acquisition, XX, YD, and QW. All authors contributed to the article and approved the submitted version.

## Funding

This study was jointly supported by research grants from Project by the Department of Science and Technology of Guangdong Province (2019QN01N107), Key Laboratory of Guangdong Province (2020B121201009), the Guangdong Province Academy of Sciences Special Project for Capacity Building of Innovation Driven Development (2020GDASYL-20200301002).The funders had no role in the design of the study, in the collection, analyses, or interpretation of data, in the writing of the manuscript, or in the decision to publish the results.

## Conflict of Interest

The authors declare that the research was conducted in the absence of any commercial or financial relationships that could be construed as a potential conflict of interest.

## Publisher’s Note

All claims expressed in this article are solely those of the authors and do not necessarily represent those of their affiliated organizations, or those of the publisher, the editors and the reviewers. Any product that may be evaluated in this article, or claim that may be made by its manufacturer, is not guaranteed or endorsed by the publisher.

## References

[B1] Birch-Machin M. A. (2000). Mitochondria and Skin Disease. Clin. Exp. Dermatol. 25(2), 141–146. doi: 10.1046/j.1365-2230.2000.00605.x 10733641

[B2] Bolger A. M. Lohse M. Usadel B. (2014). Trimmomatic: A Flexible Trimmer for Illumina Sequence Data. Bioinformatics 30(15), 2114–2120. doi: 10.1093/bioinformatics/btu170 24695404 PMC4103590

[B3] Brand R. M. Wipf P. Durham A. Epperly M. W. Greenberger J. S. Falo L. D. Jr. (2018). Targeting Mitochondrial Oxidative Stress to Mitigate UV-Induced Skin Damage. Front. Pharmacol. 9, 920. doi: 10.3389/fphar.2018.00920 30177881 PMC6110189

[B4] Cavinato M. Jansen-Dürr P. (2017). Molecular Mechanisms of UVB-Induced Senescence of Dermal Fibroblasts and its Relevance for Photoaging of the Human Skin. Exp. Gerontol. 94, 78–82. doi: 10.1016/j.exger.2017.01.009 28093316

[B5] Croucher N. J. Page A. J. Connor T. R. Delaney A. J. Keane J. A. Bentley S. D.. (2015). Rapid Phylogenetic Analysis of Large Samples of Recombinant Bacterial Whole Genome Sequences Using Gubbins. Nucleic Acids Res. 43(3), e15. doi: 10.1093/nar/gku1196 25414349 PMC4330336

[B6] Elefantova K. Lakatos B. Kubickova J. Sulova Z. Breier A. (2018). Detection of the Mitochondrial Membrane Potential by the Cationic Dye JC-1 in L1210 Cells With Massive Overexpression of the Plasma Membrane ABCB1 Drug Transporter. Int. J. Mol. Sci 19(7), 1985. doi: 10.3390/ijms19071985 PMC6073605 29986516

[B7] Fania L. Mazzanti C. Campione E. Candi E. Abeni D. Dellambra E. (2019). Role of Nicotinamide in Genomic Stability and Skin Cancer Chemoprevention. Int. J. Mol. Sci 20(23), 5946. doi: 10.3390/ijms20235946 PMC6929077 31779194

[B8] Finamore A. Ambra R. Nobili F. Garaguso I. Raguzzini A. Serafini M. (2018). Redox Role of *Lactobacillus Casei Shirota* Against the Cellular Damage Induced by 2,2’-Azobis (2-Amidinopropane) Dihydrochloride-Induced Oxidative and Inflammatory Stress in Enterocytes-Like Epithelial Cells. Front. Immunol. 9, 1131. doi: 10.3389/fimmu.2018.01131 29881384 PMC5976738

[B9] Findley K. Grice E. A. (2014). The Skin Microbiome: A Focus on Pathogens and Their Association With Skin Disease. PloS Pathog. 10(11), e1004436. doi: 10.1371/journal.ppat.1004436 25393405 PMC4231143

[B10] Friedman O. (2005). Changes Associated With the Aging Face. Facial Plast. Surg. Clin. North Am. 13(3), 371–380. doi: 10.1016/j.fsc.2005.04.004 16085282

[B11] Garrity G. M. (2016). A New Genomics-Driven Taxonomy of Bacteria and Archaea: Are We There Yet? J. Clin. Microbiol. 54(8), 1956–1963. doi: 10.1128/JCM.00200-16 27194687 PMC4963521

[B12] Giangreco A. Qin M. Pintar J. E. Watt F. M. (2008). Epidermal Stem Cells are Retained *In Vivo* Throughout Skin Aging. Aging Cell. 7(2), 250–259. doi: 10.1111/j.1474-9726.2008.00372.x 18221414 PMC2339763

[B13] Grice E. A. (2015). The Intersection of Microbiome and Host at the Skin Interface: Genomic- and Metagenomic-Based Insights. Genome Res. 25(10), 1514–1520. doi: 10.1101/gr.191320.115 26430162 PMC4579337

[B14] Han A. Chien A. L. Kang S. (2014). Photoaging. Dermatol. Clin. 32(3), 291–9, vii. doi: 10.1016/j.det.2014.03.015 24891052

[B15] Holmström K. M. Finkel T. (2014). Cellular Mechanisms and Physiological Consequences of Redox-Dependent Signalling. Nat. Rev. Mol. Cell Biol. 15(6), 411–421. doi: 10.1038/nrm3801 24854789

[B16] Kammeyer A. Luiten R. M. (2015). Oxidation Events and Skin Aging. Ageing Res. Rev. 21, 16–29. doi: 10.1016/j.arr.2015.01.001 25653189

[B17] Kim H. M. Lee D. E. Park S. D. Kim Y. T. Kim Y. J. Jeong J. W.. (2014). Oral Administration of *Lactobacillus Plantarum* HY7714 Protects Hairless Mouse Against Ultraviolet B-Induced Photoaging. J. Microbiol. Biotechnol. 24(11), 1583–1591. doi: 10.4014/jmb.1406.06038 25112318

[B18] Koh A. De Vadder F. Kovatcheva-Datchary P. Bäckhed F. (2016). From Dietary Fiber to Host Physiology: Short-Chain Fatty Acids as Key Bacterial Metabolites. Cell 165(6), 1332–1345. doi: 10.1016/j.cell.2016.05.041 27259147

[B19] Ladizinski B. McLean R. Lee K. C. Elpern D. J. Eron L. (2014). The Human Skin Microbiome. Int. J. Dermatol. 53(9), 1177–1179. doi: 10.1111/ijd.12609 25070027

[B20] Lee D. E. Huh C. S. Ra J. Choi I. D. Jeong J. W. Kim S. H.. (2015). Clinical Evidence of Effects of *Lactobacillus Plantarum* HY7714 on Skin Aging: A Randomized, Double Blind, Placebo-Controlled Study. J. Microbiol. Biotechnol. 25(12), 2160–2168. doi: 10.4014/jmb.1509.09021 26428734

[B21] Li Z. C. Bai X. Z. Peng T. W. Yi X. W. Luo L. Yang J. Z.. (2020). New Insights Into the Skin Microbial Communities and Skin Aging. Front. Microbiol. 565549. doi: 10.3389/fmicb.2020.565549 33193154 PMC7649423

[B22] Lim H. Y. Jeong D. Park S. H. Shin K. K. Hong Y. H. Kim E.. (2020). Antiwrinkle and Antimelanogenesis Effects of Tyndallized *Lactobacillus Acidophilus* KCCM12625P. Int. J. Mol. Sci 21(5), 1620. doi: 10.3390/ijms21051620 PMC7084287 32120828

[B23] Lin P. W. Myers L. E. Ray L. Song S. C. Nasr T. R. Berardinelli A. J.. (2009). *Lactobacillus Rhamnosus* Blocks Inflammatory Signaling *In Vivo via* Reactive Oxygen Species Generation. Free Radic. Biol. Med. 124(1), 1205–1211. doi: 10.1016/j.freeradbiomed.2009.07.033 PMC2760264 19660542

[B24] Liu J. Luo J. G. Ye H. Sun Y. Lu Z. X. Zeng X. X. (2009). Production, Characterization and Antioxidant Activities *In Vitro* of Exopolysaccharides From Endophytic Bacterium Paenibacillus Polymyxa EJS-3. Carbohydr. Polym. 78(2), 275–281. doi: 10.1016/j.carbpol.2009.03.046

[B25] Liu T. Song X. An Y. Wu X. Zhang W. Li J.. (2021). Lactobacillus Rhamnosus GG Colonization in Early Life Ameliorates Inflammaging of Offspring by Activating SIRT1/AMPK/PGC-1alpha Pathway. Oxid. Med. Cell. Longev. 2021, 3328505. doi: 10.1155/2021/3328505 34804363 PMC8601837

[B26] Lloyd-Price J. Arze C. Ananthakrishnan A. N. Schirmer M. Avila-Pacheco J. Poon T. W.. (2019). Multi-Omics of the Gut Microbial Ecosystem in Inflammatory Bowel Diseases. Nature 69(7758), 655–662. doi: 10.1038/s41586-019-1237-9 PMC6650278 31142855

[B27] Luna P. C. (2020). Skin Microbiome as Years Go by. Am. J. Clin. Dermatol. 21 (Suppl 1), 12–17. doi: 10.1007/s40257-020-00549-5 32910437 PMC7584528

[B28] Mai C. Qiu L. Zeng Y. Tan X. (2021). Lactobacillus Casei Strain Shirota Enhances the Ability of Geniposide to Activate SIRT1 and Decrease Inflammation and Oxidative Stress in Septic Mice. Front. Physiol. 12, 678838. doi: 10.3389/fphys.2021.678838 34616305 PMC8488262

[B29] Makrantonaki E. Eckardt R. Steinhagen-Thiessen E. Gschnell M. Zouboulis C. C. (2013). Skin Aging. MMW Fortschr. Med. 155 Spec No 2, 50–55. doi: 10.1007/s15006-013-2130-3 24734459

[B30] Mantzourani I. Kazakos S. Terpou A. Alexopoulos A. Bezirtzoglou E. Bekatorou A.. (2018). Potential of the Probiotic *Lactobacillus Plantarum* ATCC 14917 Strain to Produce Functional Fermented Pomegranate Juice. Foods 8(1), 4. doi: 10.3390/foods8010004 PMC6352242 30583502

[B31] Morita A. (2007). Tobacco Smoke Causes Premature Skin Aging. J. Dermatol. Sci. 48(3), 169–175. doi: 10.1016/j.jdermsci.2007.06.015 17951030

[B32] Naidoo K. Hanna R. Birch-Machin M. A. (2018). What is the Role of Mitochondrial Dysfunction in Skin Photoaging? Exp. Dermatol. 27(2), 124–128. doi: 10.1111/exd.13476 29197123

[B33] Nurk S. Bankevich A. Antipov D. Gurevich A. A. Korobeynikov A. Lapidus A.. (2013). Assembling Single-Cell Genomes and Mini-Metagenomes From Chimeric MDA Products. J. Comput. Biol. 20(10), 714–737. doi: 10.1089/cmb.2013.0084 24093227 PMC3791033

[B34] Owens K. Park J. H. Schuh R. Kristian T. (2013). Mitochondrial Dysfunction and NAD(+) Metabolism Alterations in the Pathophysiology of Acute Brain Injury. Transl. Stroke Res. 4(6), 618–634. doi: 10.1007/s12975-013-0278-x 24323416

[B35] Page A. J. Cummins C. A. Hunt M. Wong V. K. Reuter S. Holden M. T.. (2015). Roary: Rapid Large-Scale Prokaryote Pan Genome Analysis. Bioinformatics 31(22), 3691–3693. doi: 10.1093/bioinformatics/btv421 26198102 PMC4817141

[B36] Park J. Halliday G. M. Surjana D. Damian D. L. (2010). Nicotinamide Prevents Ultraviolet Radiation-Induced Cellular Energy Loss. Photochem. Photobiol. 86(4), 942–948. doi: 10.1111/j.1751-1097.2010.00746.x 20492562

[B37] Patra V. Gallais Sérézal I. Wolf P. (2020). Potential of Skin Microbiome, Pro- and/or Pre-Biotics to Affect Local Cutaneous Responses to UV Exposure. Nutrients 12(6), 1765. doi: 10.3390/nu12061795 PMC7353315 32560310

[B38] Poddar S. K. Sifat A. E. Haque S. Nahid N. A. Chowdhury S. Mehedi I. (2019). Nicotinamide Mononucleotide: Exploration of Diverse Therapeutic Applications of a Potential Molecule. Biomolecules 9(1), 34. doi: 10.3390/biom9010034 PMC6359187 30669679

[B39] Scharffetter-Kochanek K. Brenneisen P. Wenk J. Herrmann G. Ma W. Kuhr L.. (2000). Photoaging of the Skin From Phenotype to Mechanisms. Exp. Gerontol. 35(3), 307–316. doi: 10.1016/s0531-5565(00)00098-x 10832052

[B40] Scharschmidt T. C. Fischbach M. A. (2013). What Lives on Our Skin: Ecology, Genomics and Therapeutic Opportunities of the Skin Microbiome. Drug Discovery Today Dis. Mech 10(3-4), e83-9. doi: 10.1016/j.ddmec.2012.12.003 PMC3833721 24273587

[B41] Thompson B. C. Halliday G. M. Damian D. L. (2015). Nicotinamide Enhances Repair of Arsenic and Ultraviolet Radiation-Induced DNA Damage in HaCaT Keratinocytes and *Ex Vivo* Human Skin. PloS One 10, e0117491. doi: 10.1371/journal.pone.0117491 25658450 PMC4319842

[B42] Treangen T. J. Ondov B. D. Koren S. Phillippy A. M. (2014). The Harvest Suite for Rapid Core-Genome Alignment and Visualization of Thousands of Intraspecific Microbial Genomes. Genome Biol. 15(11), 524. doi: 10.1186/s13059-014-0524-x 25410596 PMC4262987

[B43] Varani J. Schuger L. Dame M. K. Leonard C. Fligiel S. E. G. Kang S.. (2004). Reduced Fibroblast Interaction With Intact Collagen as a Mechanism for Depressed Collagen Synthesis in Photodamaged Skin. J. Invest. Dermatol. 122(6), 1471–1479. doi: 10.1111/j.0022-202X.2004.22614.x 15175039

[B44] Vicentini F. T. He T. Shao Y. Fonseca M. J. Verri W. A. Jr. Fisher G. J.. (2011). Quercetin Inhibits UV Irradiation-Induced Inflammatory Cytokine Production in Primary Human Keratinocytes by Suppressing NF-κb Pathway. J. Dermatol. Sci. 61(3), 162–168. doi: 10.1016/j.jdermsci.2011.01.002 21282043

[B45] Watson R. E. Gibbs N. K. Griffiths C. E. Sherratt M. J. (2014). Damage to Skin Extracellular Matrix Induced by UV Exposure. Antioxid. Redox Signal. 21(7), 1063–1077. doi: 10.1089/ars.2013.5653 24124905

[B46] Wu L. E. Sinclair D. A. (2019). The Elusive NMN Transporter is Found. Nat. Metab. 1(1), 8–9. doi: 10.1038/s42255-018-0015-6 32694812

[B47] Xu C. Qiao L. Guo Y. Ma L. Cheng Y. (2018). Preparation, Characteristics and Antioxidant Activity of Polysaccharides and Proteins-Capped Selenium Nanoparticles Synthesized by Lactobacillus Casei ATCC 393. Carbohydr. Polym. 195, 576–585. doi: 10.1016/j.carbpol.2018.04.110 29805014

[B48] Yachida S. Mizutani S. Shiroma H. Shiba S. Nakajima T. Sakamoto T.. (2019). Metagenomic and Metabolomic Analyses Reveal Distinct Stage-Specific Phenotypes of the Gut Microbiota in Colorectal Cancer. Nat. Med. 25(6), 968–976. doi: 10.1038/s41591-019-0458-7 31171880

[B49] Yau Y. F. El-Nezami H. Galano J. M. Kundi Z. M. Durand T. Lee J. C. Y. (2020). *Lactobacillus rhamnosus*GG and Oat Beta-Glucan Regulated Fatty Acid Profiles Along the Gut-Liver-Brain Axis of Mice Fed With High Fat Diet and Demonstrated Antioxidant and Anti-Inflammatory Potentials. Mol. Nutr. Food Res. doi: 10.1002/mnfr.202000566 32780531

[B50] Yiasemides E. Sivapirabu G. Halliday G. M. Park J. Damian D. L. (2009). Oral Nicotinamide Protects Against Ultraviolet Radiation-Induced Immunosuppression in Humans. Carcinogenesis 30(1), 101–105. doi: 10.1093/carcin/bgn248 19028705

[B51] Ying S. Zeng D. N. Chi L. Tan Y. Galzote C. Cardona C.. (2015). The Influence of Age and Gender on Skin-Associated Microbial Communities in Urban and Rural Human Populations. PloS One 12(8), e0141842. doi: 10.1371/journal.pone.0141842 PMC4624872 26510185

[B52] Zahran W. E. Elsonbaty S. M. Moawed F. S. M. (2017). *Lactobacillus Rhamnosus* ATCC 7469 Exopolysaccharides Synergizes With Low Level Ionizing Radiation to Modulate Signaling Molecular Targets in Colorectal Carcinogenesis in Rats. Biomed. Pharmacother. 92, 384–393. doi: 10.1016/j.biopha.2017.05.089 28554134

[B53] Zeng Y. Li Y. Wu Q. P. Zhang J. M. Xie X. Q. Ding Y.. (2020). Artn 8818989. Evaluation of the Antibacterial Activity and Probiotic Potential of *Lactobacillus Plantarum* Isolated From Chinese Homemade Pickles. Can. J. Infect. Dis. Med. Microbiol. 2020, 1–11. doi: 10.1155/2020/8818989

