# Supplementary material for: Exploration of the Molecular Mechanisms Underlying the Anti-Photoaging Effect of Limosilactobacillus fermentum XJC60
Source: Front Cell Infect Microbiol. 2022 Apr 29;12:838060. doi: 10.3389/fcimb.2022.838060 (PMC9104571; doi:10.3389/fcimb.2022.838060)
Supplement: Supplementary file 1 [file Table_1.docx]

Table S1. Sequences of primers used in semiquantitative RT-PCR.

| Gene | Direction | Sequence(5’to3’) |
| --- | --- | --- |
| MMP-1 | Forward | TCTGACGTTGATCCCAGAGAGCAG |
|  | Reverse | CAGGGTGACACCAGTGACTGCAC |
| MMP-3 | Forward | TGTTAGGAGAAAGGACAGTGGTC |
|  | Reverse | CGTCACCTCCAATCCAAGGAA |
| IL-1β | Forward | AAAAGCTTGGTGATGTCTGG |
|  | Reverse | TTTCAACACGCAGGACAGG |
| IL-6 | Forward | GTGTGAAAGCAGCAAAGAGGC |
|  | Reverse | CTGGAGGTACTCTAGGTATAC |
| IL-8 | Forward | ATGATCTCCAAGCTGGGCCGTG |
|  | Reverse | TATGAATTCTCAGCCCTCTTCAAAA |
| GADPH | Forward | ACCACAGTCCATGCCATCAC |
|  | Reverse | CCACCACCCTGTTGCTGTAG |

Table S2-lactic acid bacteria isolates and their abilities of repairing UVB damage and antioxidant

| Strain ID | Strain species | Isolated Source | UV-induced HaCaT cell viability (%) | DPPH free radical scavenging rate (%) | Hydroxyl radical scavenging rate (%) |
| --- | --- | --- | --- | --- | --- |
| C5 | Lactobacillus oris | fermented food | 142.26±6.41 | 70.86±0.21 | 77.14±0.01 |
| A4 | Lactobacillus plantarum | animal faecal | 181.12±7.32 | 52.66±0.45 | 65.84±0.05 |
| C3 | Lactobacillus mucosae | fermented food | 104.32±6.46 | 55.68±0.39 | 52.46±0.03 |
| P71 | Lactobacillus reuteri | fermented food | 156.99±7.60 | 61.82±0.60 | 81.79±0.07 |
| C19 | Lactococcus lactis | fermented food | 176.51±5.64 | 58.06±0.37 | 87.19±0.04 |
| P72 | Lactobacillus plantarum | fermented food | 149.60±2.52 | 71.78±0.37 | 82.38±0.04 |
| XJC60 | Limosilactobacillus fermentum | fermented food | 120.10±3.25 | 85.13±0.34 | 84.14±0.03 |
| C65 | Limosilactobacillus fermentum | fermented food | 139.49±1.79 | 56.13±0.43 | 94.71±0.06 |
| P69 | Lactobacillus oris | fermented food | 137.96±0.32 | 69.42±0.11 | 63.05±0.12 |
| P87 | Lactobacillus oris | fermented food | 132.98±9.39 | 66.62±0.21 | 68.16±0.02 |
| C64 | Limosilactobacillus fermentum | fermented food | 157.46±2.62 | 72.55±0.36 | 90.70±0.05 |
| C15 | Lactobacillus plantarum | fermented food | 153.30±1.18 | 66.33±0.19 | 75.27±0.03 |
| C89 | Lactobacillus plantarum | fermented food | 110.36±0.22 | 73.54±0.27 | 75.13±0.02 |
| C80 | Limosilactobacillus fermentum | fermented food | 125.79±4.29 | 60.99±0.17 | 91.86±0.04 |
| A29 | Lactobacillus brevis | animal faecal | 189.40±8.52 | 76.42±0.14 | 87.14±0.03 |
| A15 | Lactobacillus plantarum | animal faecal | 139.16±9.48 | 69.97±0.22 | 84.25±0.04 |
| R61 | Lactobacillus rhamnosus | fermented food | 135.58±6.3 | 63.83±0.31 | 81.61±0.08 |
| A27 | Lactobacillus oris | animal faecal | 140.45±0.61 | 52.04±0.37 | 44.29±0.06 |
| P76 | Lactobacillus plantarum | fermented food | 173.39±1.40 | 66.62±0.45 | 68.16±0.02 |
| C48 | Lactobacillus plantarum | fermented food | 132.87±6.91 | 83.30±0.48 | 74.02±0.12 |
| C95 | Lactobacillus plantarum | fermented food | 159.00±5.99 | 64.70±0.38 | 77.43±0.06 |
| A28 | Lactobacillus equi | animal faecal | 123.55±5.93 | 53.62±0.35 | 78.17±0.08 |
| A33 | Lactococcus lactis | animal faecal | 118.60±2.41 | 55.71±0.18 | 78.97±0.08 |
| A5 | Lactococcus lactis | animal faecal | 162.98±0.73 | 50.35±0.30 | 71.57±0.02 |
| A38 | Lactococcus lactis | animal faecal | 119.56±5.18 | 52.18±0.24 | 71.00±0.06 |
| R39 | Lactobacillus plantarum | fermented food | 117.93±4.15 | 65.8±0.26 | 69.93±0.03 |
| R60 | Lactobacillus rhamnosus | fermented food | 113.43±6.40 | 56.65±0.18 | 73.48±0.01 |
| A48 | Lactobacillus equi | animal faecal | 117.52±8.3 | 58.59±0.13 | 68.52±0.04 |
| A40 | Lactobacillus equi | animal faecal | 109.36±1.14 | 60.06±0.22 | 89.13±0.02 |
| R63 | Lactobacillus plantarum | fermented food | 131.34±0.5 | 50.74±0.38 | 95.97±0.08 |
| shirota | Lactobacillus casei | fermented food | 128.90±2.37 | 46.89±0.20 | 56.17±0.09 |
| ATCC_53103 | Lactobacillus plantarum | ATCC | 102.23±0.46 | 60.07±0.26 | 46.00±0.06 |
| C69 | Lactobacillus plantarum | fermented food | 86.22±5.77 | 20.38±0.12 | 42.71±0.03 |
| A43 | Lactobacillus equi | animal faecal | 85.71±6.99 | 29.89±0.30 | 58.16±0.01 |
| C2 | Lactococcus lactis | fermented food | 87.31±3.73 | 46.32±0.13 | 47.21±0.26 |
| C79 | Lactobacillus brevis | fermented food | 74.87±11.96 | 42.45±0.12 | 59.77±0.11 |
| C38 | Lactobacillus garvieae | fermented food | 71.72±8.13 | 48.65±0.35 | 50.23±0.06 |
| C41 | Limosilactobacillus fermentum | fermented food | 69.85±3.14 | 20.82±0.21 | 55.54±0.04 |
| P68 | Lactobacillus brevis | fermented food | 69.13±3.22 | 55.93±0.34 | 58.85±0.03 |
| G34 | Lactobacillus plantarum | animal faecal | 68.44±8.59 | 28.69±0.18 | 36.93±0.02 |
| R58 | Lactobacillus paracasei | fermented food | 67.36±7.98 | 46.89±0.07 | 56.17±0.02 |
| A20 | Lactobacillus plantarum | animal faecal | 61.80±5.98 | 46.42±0.26 | 54.33±0.01 |
| A3 | Lactobacillus reuteri | animal faecal | 61.77±2.40 | 42.64±0.20 | 54.18±0.01 |
| Q64 | Lactobacillus plantarum | fermented food | 93.54±4.68 | 36.51±0.09 | 42.98±0.02 |
| C70 | Lactococcus lactis | fermented food | 58.90±3.23 | 28.62±0.20 | 43.90±0.01 |
| P27 | Limosilactobacillus fermentum | fermented food | 58.67±7.94 | 28.13±0.31 | 57.51±0.03 |
| P94 | Lactobacillus plantarum | fermented food | 56.63±3.81 | 26.36±0.08 | 52.29±0.15 |
| A42 | Lactobacillus brevis | animal faecal | 52.4±5.37 | 56.12±0.12 | 47.50±0.21 |
| C34 | Lactobacillus gallinarum | fermented food | 51.8±1.45 | 41.75±0.30 | 50.96±0.03 |
| Q38 | Lactobacillus saluzvarius | fermented food | 51.48±9.76 | 40.91±0.15 | 57.28±0.11 |
| Q49 | Lactobacillus plantarum | fermented food | 80.76±7.90 | 41.81±0.17 | 58.51±0.20 |
| Q56 | Lactococcus lactis | fermented food | 79.76±9.27 | 21.91±0.23 | 65.94±0.18 |
| Q47 | Lactobacillus plantarum | fermented food | 45.60±3.82 | 26.98±0.17 | 50.84±00.01 |
| C22 | Lactobacillus rhamnosus | animal faecal | 67.89±5.14 | 27.57±0.10 | 41.04±0.02 |
| Q40 | Lactobacillus fermantum | fermented food | 30.44±3.26 | 61.55±0.22 | 64.44±0.10 |
| K92 | Lactobacillus reuteri | animal faecal | 28.42±2.46 | 74.46±0.15 | 58.66±0.10 |
| A53 | Lactococcus lactis | animal faecal | 27.10±5.54 | 48.86±0.13 | 35.17±0.06 |
| Q6 | Lactobacillus plantarum | fermented food | 24.79±3.83 | 54.63±0.06 | 47.04±0.11 |
| C68 | Lactobacillus plantarum | fermented food | 55.76±6.69 | 40.44±0.05 | 43.70±0.19 |
| R20 | Lactobacillus plantarum | fermented food | 20.56±1.46 | 26.71±0.06 | 56.83±0.14 |
| Q11 | Lactobacillus plantarum | fermented food | 53.80±8.36 | 38.56±0.27 | 46.54±0.00 |
| P38 | Limosilactobacillus fermentum | fermented food | 86.95±2.87 | 47.90±0.17 | 53.9±0.14 |
| P82 | Lactobacillus plantarum | fermented food | 80.32±2.13 | 24.27±0.25 | 87.55±0.05 |
| A50 | Lactobacillus equi | animal faecal | 83.63±6.96 | 52.91±0.10 | 53.94±0.01 |
| Q30 | Limosilactobacillus fermentum | fermented food | 60.70±4.61 | 32.36±0.03 | 66.28±0.06 |
| Q5 | Lactococcus lactis | fermented food | 58.28±8.94 | 42.98±0.08 | 46.79±0.04 |
| A30 | Lactobacillus plantarum | animal faecal | 71.77±5.12 | 24.27±0.44 | 50.30±0.10 |
| C62 | Lactococcus lactis | fermented food | 96.70±3.98 | 40.75±0.46 | 52.51±0.05 |
| A51 | Lactococcus garvieae | animal faecal | 95.21±8.46 | 55.74±0.77 | 52.27±0.02 |
| Q34 | Limosilactobacillus fermentum | fermented food | 92.13±1.32 | 35.80±0.90 | 39.93±0.05 |
| C92 | Limosilactobacillus fermentum | fermented food | 92.72±6.44 | 37.10±0.57 | 37.77±0.08 |
| C54 | Limosilactobacillus fermentum | fermented food | 91.56±9.16 | 36.51±0.65 | 42.98±0.08 |
| C17 | Limosilactobacillus fermentum | fermented food | 89.45±5.26 | 50.24±0.32 | 55.52±0.03 |
| A65 | Lactobacillus equi | fermented food | 87.68±9.22 | 52.59±0.25 | 51.94±0.18 |
| A11 | Lactobacillus equi | animal faecal | 77.34±6.50 | 34.35±0.68 | 49.84±0.21 |
| Q74 | Lactobacillus plantarum | fermented food | 84.10±9.90 | 48.92±0.45 | 58.33±0.03 |
| Q63 | Lactobacillus plantarum | fermented food | 80.65±7.47 | 60.95±0.34 | 64.99±0.02 |
| C53 | Limosilactobacillus fermentum | fermented food | 77.44±4.3 | 54.79±0.20 | 50.58±0.13 |
| P44 | Limosilactobacillus fermentum | fermented food | 76.93±4.41 | 44.32±0.65 | 42.51±0.11 |
| G39 | Limosilactobacillus fermentum | animal faecal | 74.49±3.77 | 44.59±0.51 | 51.29±0.02 |
| R54 | Lactobacillus plantarum | fermented food | 73.51±6.38 | 36.67±0.35 | 52.88±0.15 |
| Q25 | Limosilactobacillus fermentum | fermented food | 73.67±7.40 | 22.46±0.05 | 57.63±0.23 |
| PV5 | Limosilactobacillus fermentum | animal faecal | 76.42±9.76 | 35.76±0.11 | 44.38±0.39 |
| P42 | Lactobacillus plantarum | fermented food | 70.60±9.98 | 47.31±0.21 | 54.38±0.11 |
| Q62 | Lactobacillus plantarum | fermented food | 69.38±6.14 | 52.91±0.33 | 56.38±0.01 |
| Q10 | Lactobacillus reuteri | fermented food | 69.34±5.30 | 55.24±0.16 | 60.94±0.02 |
| A14 | Lactobacillus equi | animal faecal | 68.48±8.32 | 41.98±0.31 | 40.72±0.06 |
| A32 | Lactobacillus garvieae | animal faecal | 64.65±1.22 | 68.60±0.39 | 45.89±0.02 |
| R31 | Lactobacillus rhamnosus | fermented food | 62.20±0.52 | 24.72±0.06 | 59.19±0.01 |
| R53 | Lactobacillus plantarum | fermented food | 61.18±7.72 | 43.18±0.19 | 62.83±0.07 |
| R59 | Lactobacillus plantarum | fermented food | 60.32±6.12 | 24.25±0.29 | 59.64±0.02 |
| R36 | Lactobacillus mucosae | fermented food | 59.47±8.78 | 31.48±0.96 | 46.53±0.05 |
| A12 | Lactobacillus saluzvarius | animal faecal | 58.88±6.58 | 50.79±1.41 | 71.34±0.04 |
| C42 | Lactobacillus plantarum | animal faecal | 56.19±8.16 | 27.28±0.52 | 38.79±0.04 |
| P15 | Lactobacillus plantarum | fermented food | 53.54±3.94 | 41.31±0.07 | 56.38±0.05 |
| Q65 | Lactobacillus plantarum | fermented food | 52.85±1.82 | 46.57±0.11 | 45.13±0.03 |
| R64 | Lactiplantibacillus plantarum | fermented food | 50.15±6.17 | 28.50±0.29 | 46.99±0.04 |
| Q36 | Limosilactobacillus fermentum | fermented food | 50.13±1.44 | 41.58±0.14 | 35.90±0.14 |
| Q32 | Limosilactobacillus fermentum | fermented food | 50.42±6.24 | 36.97±0.16 | 52.61±0.13 |
| G38 | Limosilactobacillus fermentum | animal faecal | 77.00±2.31 | 23.53±0.22 | 52.63±0.06 |
| A49 | Lactobacillus plantarum | animal faecal | 75.89±1.67 | 63.44±0.16 | 52.86±0.08 |
| PV20 | Limosilactobacillus fermentum | animal faecal | 74.61±8.95 | 21.25±0.09 | 49.09±0.06 |
| C36 | Lactobacillus equi | fermented food | 73.10±9.85 | 51.69±0.21 | 45.89±0.04 |
| PV18 | Limosilactobacillus fermentum | animal faecal | 72.33±11.62 | 21.88±0.14 | 40.79±0.04 |
| PV38 | Lactobacillus saluzvarius | animal faecal | 70.54±9.11 | 25.83±0.12 | 47.13±0.06 |
| G43 | Lactobacillus plantarum | animal faecal | 69.30±8.34 | 50.79±0.05 | 71.34±0.06 |
| R52 | Lactobacillus paracasei | fermented food | 67.57±3.59 | 26.05±0.04 | 50.13±0.03 |
| P41 | Lactobacillus plantarum | fermented food | 64.38±2.72 | 31.90±0.05 | 51.12±0.01 |
| R57 | Lactobacillus paracasei | fermented food | 64.49±7.24 | 39.96±0.26 | 54.46±0.05 |
| P30 | Limosilactobacillus fermentum | fermented food | 64.15±5.52 | 25.74±0.16 | 50.66±0.06 |
| PV35 | Lactobacillus saluzvarius | animal faecal | 64.36±5.72 | 43.55±0.24 | 35.86±0.13 |
| C23 | Lactobacillus garvieae | fermented food | 83.86±3.98 | 60.78±0.09 | 46.37±0.16 |
| Q71 | Lactococcus lactis | fermented food | 79.56±16.56 | 34.62±0.02 | 43.31±0.03 |
| P61 | Lactobacillus debrueckii | fermented food | 78.32±18.28 | 30.31±0.07 | 58.13±0.02 |
| Q19 | Limosilactobacillus fermentum | fermented food | 77.67±19.5 | 37.63±0.43 | 84.58±0.03 |
| G21 | Lactobacillus equi | animal faecal | 76.56±13.56 | 57.74±0.18 | 49.08±0.03 |
| C50 | Limosilactobacillus fermentum | fermented food | 75.49±17.24 | 43.24±0.17 | 46.52±0.05 |
| P1 | Limosilactobacillus fermentum | fermented food | 73.18±15.65 | 22.04±0.40 | 14.29±0.01 |
| P51 | Limosilactobacillus fermentum | fermented food | 70.49±16.30 | 39.97±0.26 | 52.32±0.03 |
| A41 | Lactobacillus plantarum | animal faecal | 68.54±7.29 | 43.59±0.39 | 42.57±0.02 |
| Q17 | Lactobacillus plantarum | fermented food | 68.33±12.55 | 65.40±0.23 | 49.25±0.11 |
| C27 | Limosilactobacillus fermentum | fermented food | 67.33±8.69 | 23.39±0.12 | 29.35±0.02 |
| C55 | Lactobacillus rhamnosus | fermented food | 67.9±5.48 | 45.32±0.31 | 50.12±0.03 |
| C11 | Lactobacillus plantarum | fermented food | 66.76±8.80 | 21.44±0.25 | 55.33±0.01 |
| C9 | Lactobacillus plantarum | fermented food | 66.91±12.34 | 51.08±0.14 | 49.06±0.03 |
| G33 | Lactobacillus reuteri | animal faecal | 65.21±10.61 | 37.45±0.25 | 53.88±0.07 |
| G25 | Limosilactobacillus fermentum | animal faecal | 64.54±9.28 | 60.72±0.36 | 59.32±0.04 |
| Q45 | Lactobacillus plantarum | fermented food | 63.94±10.74 | 53.31±0.13 | 44.29±0.02 |
| C52 | Limosilactobacillus fermentum | fermented food | 62.45±12.87 | 20.06±0.17 | 42.07±0.02 |
| Q70 | Lactobacillus plantarum | fermented food | 61.47±16.5 | 26.75±0.35 | 48.6±0.03 |
| C58 | Lactobacillus garvieae | fermented food | 59.84±16.32 | 66.49±0.21 | 63.36±0.01 |
| C25 | Lactobacillus plantarum | fermented food | 58.72±17.30 | 68.30±0.22 | 53.24±0.01 |
| Q55 | Lactobacillus plantarum | fermented food | 55.54±9.19 | 54.09±0.28 | 62.58±0.02 |
| Q51 | Lactobacillus plantarum | fermented food | 55.7±14.29 | 44.45±0.22 | 58.74±0.01 |
| R25 | Lactococcus lactis | fermented food | 55.7±19.76 | 27.29±0.15 | 66.63±0.03 |
| A2 | Lactobacillus equi | animal faecal | 52.4±8.45 | 40.44±0.27 | 53.31±0.01 |
| G32 | Lactobacillus brevis | animal faecal | 52.78±7.78 | 76.24±0.20 | 75.68±0.04 |
| C44 | Lactobacillus garvieae | fermented food | 50.41±6.57 | 42.84±0.18 | 54.96±0.12 |
| R49 | Lactobacillus mucosae | animal faecal | 69.18±7.80 | 22.37±0.11 | 56.08±0.05 |
| C32 | Limosilactobacillus fermentum | fermented food | 69.81±10.73 | 32.57±0.10 | 35.86±0.05 |
| K21 | Lactobacillus reuteri | animal faecal | 67.36±14.65 | 38.99±0.11 | 41.88±0.09 |
| Q57 | Lactobacillus plantarum | animal faecal | 67.62±10.89 | 48.75±0.32 | 54.87±0.03 |
| C20 | Lactobacillus debrueckii | fermented food | 65.33±12.27 | 24.39±0.22 | 30.08±0.04 |
| A31 | Lactococcus lactis | animal faecal | 62.97±13.55 | 36.17±0.35 | 43.01±0.02 |
| R46 | Lactobacillus plantarum | fermented food | 60.97±8.71 | 33.09±0.15 | 53.25±0.03 |
| Q31 | Lactobacillus plantarum | animal faecal | 60.8±10.48 | 38.50±0.08 | 41.18±0.01 |
| R7 | Lactobacillus mucosae | fermented food | 60.22±3.64 | 43.54±0.13 | 45.13±0.03 |
| C4 | Lactobacillus reuteri | fermented food | 57.37±15.59 | 62.76±0.49 | 60.15±0.17 |
| G49 | Lactobacillus equi | animal faecal | 56.25±2.55 | 53.98±0.51 | 55.86±0.04 |
| C75 | Lactobacillus debrueckii | fermented food | 55.15±6.68 | 42.04±0.82 | 72.37±0.02 |
| C26 | Limosilactobacillus fermentum | fermented food | 54.74±5.52 | 38.56±0.95 | 46.54±0.04 |
| K94 | Lactobacillus equi | animal faecal | 54.74±2.1 | 62.19±0.62 | 56.47±0.03 |
| R38 | Limosilactobacillus fermentum | fermented food | 53.15±3.59 | 22.99±0.70 | 50.46±0.06 |
| C47 | Lactococcus lactis | fermented food | 52.74±6.92 | 52.50±0.37 | 55.69±0.07 |
| C56 | Lactobacillus plantarum | fermented food | 52.56±5.63 | 46.99±0.35 | 35.25±0.19 |
| C1 | Lactobacillus plantarum | fermented food | 49.58±3.71 | 21.99±0.73 | 45.28±0.20 |
| G18 | Lactobacillus plantarum | animal faecal | 48.70±10.76 | 77.10±0.50 | 67.10±0.03 |
| G36 | Limosilactobacillus fermentum | animal faecal | 47.55±9.90 | 35.93±0.39 | 45.64±0.10 |
| Q53 | Limosilactobacillus fermentum | fermented food | 46.25±5.70 | 20.51±0.25 | 55.52±0.07 |
| C59 | Limosilactobacillus fermentum | fermented food | 45.4±10.80 | 23.24±0.70 | 45.72±0.17 |
| C57 | Limosilactobacillus fermentum | fermented food | 45.11±3.47 | 27.94±0.56 | 39.50±0.19 |
| Q68 | Lactobacillus plantarum | fermented food | 39.80±4.33 | 39.84±0.40 | 49.60±0.06 |
| C91 | Lactobacillus plantarum | fermented food | 38.81±8.55 | 21.76±0.45 | 62.99±0.16 |
| PV12 | Limosilactobacillus fermentum | animal faecal | 36.13±0.87 | 20.27±0.47 | 45.12±0.13 |
| Q80 | Lactobacillus debrueckii | fermented food | 32.74±3.18 | 26.35±0.78 | 40.25±0.04 |
| R96 | Lactobacillus plantarum | animal faecal | 60.80±6.21 | 22.41±0.91 | 47.17±0.04 |
| R95 | Lactobacillus plantarum | animal faecal | 86.89±26.50 | 64.80±0.58 | 54.06±0.07 |
| ATCC_14917 | Lactobacillus plantarum | ATCC | 84.34±15.99 | 68.42±0.66 | 52.37±0.07 |
| K22 | Lactobacillus equi | animal faecal | 78.45±10.85 | 29.01±0.33 | 58.87±0.08 |
| P50 | Lactobacillus equi | animal faecal | 77.28±9.65 | 25.42±0.26 | 45.94±0.06 |
| ATCC_393 | Lactobacillus casei | ATCC | 76.40±15.76 | 63.04±0.69 | 58.43±0.08 |
| PV11 | Limosilactobacillus fermentum | human faecal | 69.88±4.77 | 56.99±0.46 | 60.07±0.06 |
| R33 | Limosilactobacillus fermentum | fermented food | 66.11±13.84 | 41.22±0.35 | 34.05±0.07 |
| PV68 | Lactobacillus mucosae | human faecal | 62.94±12.52 | 41.73±0.21 | 41.90±0.02 |
| PV63 | Lactobacillus mucosae | human faecal | 45.36±5.81 | 32.73±0.66 | 36.62±0.07 |
| A69 | Lactobacillus mucosae | fermented food | 76.10±13.75 | 43.84±0.52 | 42.92±0.02 |
| C14 | Lactobacillus reuteri | fermented food | 95.11±4.6 | 26.68±0.36 | 53.10±0.03 |
| PV70 | Lactobacillus gallinarum | human faecal | 96.87±5.7 | 37.80±0.06 | 38.53±0.04 |
| C28 | Lactobacillus gallinarum | fermented food | 94.73±15.36 | 38.99±0.12 | 41.88±0.01 |
| R34 | Lactobacillus debrueckii | animal faecal | 88.75±10.67 | 28.60±0.22 | 50.26±0.09 |
| R6 | Lactobacillus debrueckii | animal faecal | 87.58±9.15 | 34.53±0.34 | 39.03±0.01 |
| P13 | Lactobacillus debrueckii | animal faecal | 86.28±15.36 | 62.90±0.17 | 59.43±0.05 |
| A26 | Lactobacillus garvieae | fermented food | 79.13±4.49 | 31.12±0.31 | 55.53±0.03 |
| A67 | Lactobacillus equi | fermented food | 76.11±13.47 | 52.45±0.40 | 49.84±0.04 |
| PV46 | Lactobacillus equi | human faecal | 72.35±12.61 | 24.25±0.07 | 60.15±0.08 |
| K96 | Lactobacillus saluzvarius | animal faecal | 55.7±5.54 | 22.81±0.20 | 49.87±0.06 |
| PV44 | Lactobacillus saluzvarius | human faecal | 70.26±15.20 | 41.11±0.30 | 45.49±0.02 |
| PV19 | Lactobacillus paracasei | human faecal | 70.23±9.18 | 38.37±0.97 | 63.09±0.02 |
| PV65 | Lactobacillus paracasei | human faecal | 70.11±6.87 | 43.46±1.42 | 52.97±0.04 |
| R62 | Lactobacillus rhamnosus | animal faecal | 70.68±17.63 | 62.63±0.51 | 62.31±0.01 |
| PV54 | Lactobacillus rhamnosus | human faecal | 67.21±15.87 | 53.85±0.08 | 58.47±0.02 |
| PV47 | Lactobacillus rhamnosus | human faecal | 67.43±13.75 | 41.91±0.12 | 66.36±0.03 |
| PV50 | Lactobacillus rhamnosus | human faecal | 66.64±5.92 | 38.43±0.30 | 53.05±0.03 |
| PV78 | Lactobacillus brevis | human faecal | 65.39±2.86 | 62.06±0.15 | 75.41±0.02 |
| A47 | Lactobacillus plantarum | animal faecal | 65.5±6.15 | 22.85±0.17 | 54.69±0.05 |
| R51 | Lactobacillus plantarum | fermented food | 64.16±5.74 | 52.37±0.23 | 55.81±0.02 |
| R19 | Lactobacillus plantarum | fermented food | 64.9±15.0 | 46.86±0.17 | 35.66±0.01 |
| R32 | Lactobacillus plantarum | fermented food | 63.86±10.44 | 21.86±0.10 | 41.61±0.03 |
| PV66 | Lactobacillus plantarum | human faecal | 62.85±6.78 | 76.97±0.22 | 54.61±0.02 |
| PV74 | Lactobacillus plantarum | human faecal | 62±15.56 | 35.79±0.15 | 29.81±0.07 |
| PV55 | Lactobacillus plantarum | human faecal | 59.11±17.33 | 20.37±0.13 | 42.73±0.04 |
| PV49 | Lactobacillus plantarum | human faecal | 58.56±10.64 | 23.10±0.06 | 52.98±0.04 |
| R17 | Lactobacillus plantarum | animal faecal | 57.40±9.68 | 27.81±0.05 | 40.92±0.04 |
| C21 | Limosilactobacillus fermentum | fermented food | 56.18±15.3 | 39.71±0.06 | 44.86±0.01 |
| ATCC_7469 | Lactobacillus plantarum | ATCC | 55.68±10.84 | 21.62±0.27 | 59.88±0.06 |
| P14 | Lactobacillus equi | animal faecal | 55.31±3.20 | 20.14±0.17 | 55.59±0.05 |
